# ADAM-Net: Anatomy-Guided Attentive Unsupervised Domain Adaptation for Joint MG Segmentation and MGD Grading

**DOI:** 10.3390/jimaging12010050

**Published:** 2026-01-21

**Authors:** Junbin Fang, Xuan He, You Jiang, Mini Han Wang

**Affiliations:** 1 Department of Optoelectronic Engineering, Jinan University, Guangzhou 510632, China; hexuan@stu2023.jnu.edu.cn (X.H.); youjiang@stu2022.jnu.edu.cn (Y.J.); 2 Guangdong Provincial Key Laboratory of Optical Fiber Sensing and Communications, Guangzhou 510632, China; 3 Guangdong Provincial Engineering Technology Research Center on Visible Light Communication, Guangzhou 510632, China; 4 Guangzhou Municipal Key Laboratory of Engineering Technology on Visible Light Communication, Guangzhou 510632, China; 5 Faculty of Medicine, Chinese University of Hong Kong, Hong Kong 999077, China

**Keywords:** domain adaptation, multi-task learning, generalization, classification, segmentation

## Abstract

Meibomian gland dysfunction (MGD) is a leading cause of dry eye disease, assessable through gland atrophy degree. While deep learning (DL) has advanced meibomian gland (MG) segmentation and MGD classification, existing methods treat these tasks independently and suffer from domain shift across multi-center imaging devices. We propose ADAM-Net, an attention-guided unsupervised domain adaptation multi-task framework that jointly models MG segmentation and MGD classification. Our model introduces structure-aware multi-task learning and anatomy-guided attention to enhance feature sharing, suppress background noise, and improve glandular region perception. For the cross-domain tasks MGD-1K→{K5M, CR-2, LV II}, this study systematically evaluates the overall performance of ADAM-Net from multiple perspectives. The experimental results show that ADAM-Net achieves classification accuracies of 77.93%, 74.86%, and 81.77% on the target domains, significantly outperforming current mainstream unsupervised domain adaptation (UDA) methods. The F1-score and the Matthews correlation coefficient (MCC-score) indicate that the model maintains robust discriminative capability even under class-imbalanced scenarios. t-SNE visualizations further validate its cross-domain feature alignment capability. These demonstrate that ADAM-Net exhibits strong robustness and interpretability in multi-center scenarios and provide an effective solution for automated MGD assessment.

## 1. Introduction

MGD is a common ocular surface disease characterized by obstruction or atrophy of the MG [1]. Clinically, the severity of MGD is assessed based on infrared Meibography by analyzing indicators such as MG dropout area, length, and others [2]. Among these, the dropout area is the most critical parameter [3]. MGD is usually graded according to the ratio of dropout area to total eyelid area [4]. Traditional clinical diagnosis relies on physicians’ manual experience, which suffers from strong subjectivity, low inter- and intra-observer reproducibility, and low efficiency [5,6].

Image processing and machine learning (ML) techniques have been introduced into automated MG analysis [3,7,8,9,10,11]. Arita et al. developed a semi-automatic MG assessment method using Image J (National Institutes of Health, Bethesda, Rockville, ML, USA), which quantified gland atrophy through manual annotation [3]. Llorens-Quintana et al. combined threshold segmentation, morphological processing, and curve fitting to create an automated framework for MG segmentation and irregularity quantification, allowing for atrophy estimation [7,8]. Koh et al. employed Gaussian smoothing, morphological processing, and SIFT Shannon entropy to extract MG dimensions, then trained a linear Support Vector Machine (SVM) classifier to assess gland health. This represented an early application of supervised ML to Meibography assessment [9]. Cięzar et al. proposed an innovative frequency-domain analysis method based on the 2D Fourier transform, which enables pathological assessment through classifiers directly utilizing frequency-domain features without requiring gland segmentation [10]. While these methods perform well on high-quality images, they struggle with blurred gland boundaries or uneven illumination, often requiring manual region of interest (ROI) selection. This limits their clinical utility and efficiency [11].

DL methods greatly improve diagnostic efficiency and accuracy through end-to-end feature learning, emerging as the main approach for automated MG analysis [12]. Current Meibography research mainly develops: (1) semantic segmentation models for MG and eyelid region extraction [6,13,14,15] and (2) classification models for automated MGD grading [12,16,17]. In segmentation, Wang et al. employed PSPNet to achieve global segmentation of the eyelid and atrophy regions, although without resolving individual glands [6]. Prabhu et al. pioneered the application of the U-Net architecture for MG segmentation [13]. Setu et al. integrated transfer learning with U-Net in a unified framework for both upper and lower eyelids, achieving automatic gland segmentation with an 84% Dice score despite gland adhesion [14]. Zhu et al. proposed the SBD-MTLNet multi-task network, which further increased the Dice coefficient to 84.32% through strip attention and boundary detection modules [15]. In classification, Luo et al. achieved 94% accuracy with an AlexNet-based model but faced interpretability limitations [12]. Wang et al. improved classification accuracy from 67% to 86% using explainable AI (XAI)-guided image correction [16]. Yeh et al. used unsupervised instance discrimination to achieve 80.9% accuracy with reduced annotation needs, though remaining sensitive to image quality [17]. Although they have made progress in segmentation and classification, most studies still handle them separately, neglecting their intrinsic collaboration. In fact, MG segmentation provides morphological details crucial for atrophy assessment, while MGD classification offers pathological context for segmentation. Isolated modeling prevents cross-task feature exchange, compromising both discriminative power and clinical interpretability. To address this limitation, Saha et al. implemented a cascaded approach that first segments gland regions and then uses them for grade prediction, leveraging structural information to assist assessment [11]. However, this method remains a “fixed prior” with one-way dependency, which limits task collaboration. In contrast, multi-task learning enables simultaneous MG segmentation and MGD classification through shared feature extraction. This promotes inter-task collaboration during training, enhancing structural comprehension of gland regions and improving both diagnostic precision and clinical interpretability.

Although Saha et al. achieved 73.01% accuracy on same-center test data, their performance dropped to 59.17% when evaluated on external center data [11]. The performance degradation results from the violation of the i.i.d. assumption in DL [18]. Especially in multi-center medical studies due to limitations such as different imaging devices and parameters, there are significant distribution differences between datasets, i.e., domain shift [19]. The test error typically increases with the magnitude of the domain shift [20]. While traditional supervised learning fits source domains well, its target domain generalization often drops significantly, limiting multi-center clinical deployment. Domain adaptation (DA) addresses this by reducing source-target domain distribution differences and aligning feature spaces to improve target domain generalization [21]. UDA is particularly notable, as it does not require target domain labels.

Model generalization is influenced not only by domain shift but also by image quality and the associated noise characteristics, which represent equally important limiting factors. It should be noted that the decline in generalization performance is not solely due to variations in the signal-to-noise ratio (SNR) but more critically to differences in the noise models themselves [22]. When test data exhibits similar noise statistics to training data, the model may maintain good generalization performance even with variations in the SNR. However, when the noise type or distribution changes, even if the overall image quality or SNR level remains similar, the model may still experience a significant decline in generalization ability due to shifts in noise characteristics. Clinically acquired Meibography often contains background interference (e.g., eyelashes, eyeballs). Such non-structural noise impairs accurate MG region recognition [23]. During DA, models may exploit background interference as domain-discriminative cues, extracting biased features that hinder cross-domain alignment and generalization. Integrating attention mechanisms helps in focusing on the ROI while suppressing irrelevant information, improving both structural discrimination and cross-domain performance [24].

**Figure 1:**
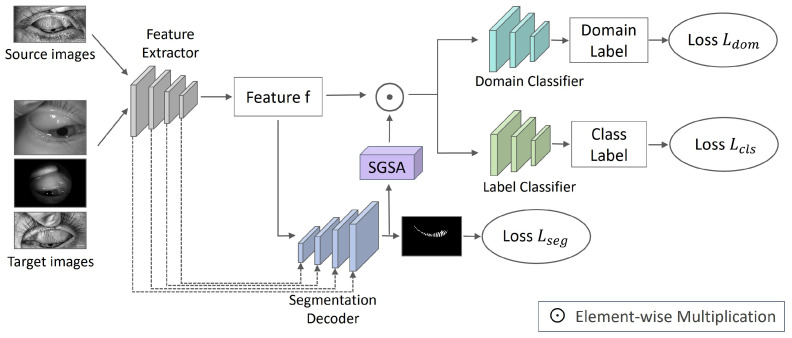
Overall architecture of the proposed ADAM-Net for attention-guided multi-task unsupervised domain adaptation.

To address the challenges mentioned above, this paper proposes an attention-guided unsupervised domain-adaptive multi-task network for Meibography analysis, called ADAM-Net. The framework is illustrated in Figure 1. This method systematically enhances model performance from three aspects: domain adaptation, multi-task learning, and attention mechanism. (1) To mitigate performance degradation caused by domain shift in multi-center data, this study builds upon the Domain Adversarial Neural Network (DANN) [25]. By aligning source and target feature distributions via adversarial learning, the proposed approach enhances cross-domain generalization under unsupervised conditions. (2) This study also aims to overcome the limitations of modeling MG segmentation and MGD classification independently, which restricts feature sharing and increases computational overhead. This study introduces a multi-task learning strategy that integrates MG segmentation and MGD classification tasks into a unified framework. This allows the model to extract shared features while retaining task-specific information [26]. Furthermore, an uncertainty-weighted strategy adaptively balances task losses, enabling collaborative optimization with reduced computational overhead [27]. This effectively alleviates the performance bottleneck caused by independent task modeling. (3) Another aim is to reduce the impact of background noise on ROI perception, thereby affecting feature discriminability and domain alignment. This study proposes a Segmentation-Guided Spatial Attention (SGSA) module that generates spatial attention maps using MG segmentation masks. These maps dynamically guide the classification branch and the domain discriminator to focus on the MG region. A consistency loss is used to regularize and strengthen spatial structural consistency between tasks. This helps suppress background noise and enhance the model’s perception of the ROI. It also reduces the misleading influence of non-semantic regions on the domain discriminator, thereby improving the effectiveness of domain alignment.

Our contributions are summarized as follows: (1) We propose a UDA multi-task network framework, which aligns feature distributions through domain adversarial learning constructed by a domain discriminator and a gradient reversal layer (GRL). An uncertainty-based weighted strategy is employed to jointly optimize the classification and segmentation tasks, thus facilitating dual-task knowledge transfer. This improves classification performance on unlabeled target domains and generalization of the model. (2) We propose the SGSA module, which leverages segmentation masks to enhance spatial attention. Combined with a consistency loss for regularization, this module suppresses background noise and strengthens the model’s focus on the ROI, thereby improving cross-domain feature alignment.

To better position the novelty of the proposed method, Table 1 provides a structured overview of representative methods and their modeling capabilities.

## 2. Methods

This section introduces the proposed ADAM-Net model, which performs MG segmentation and MGD classification while aligning the feature distributions between the source and target domains. The design of the model’s key modules and loss functions will be described in detail.

We first define the source domain dataset as: $D_{s}={\left\{\left(x_{i}^{s},y_{i}^{s},m_{i}^{s}\right)\right\}}_{i=1}^{n_{s}}$, which contains $n_{s}$ labeled samples $x_{i}^{s}$ with Meiboscore labels $y_{i}^{s}$ and MG segmentation masks $m_{i}^{s}$; and the target domain dataset $D_{t}={\left\{x_{j}^{t}\right\}}_{j=1}^{n_{t}}$, which contains $n_{t}$ unlabeled samples $x_{j}^{t}$. The source and target domain data are drawn from joint distributions $P(x_{s},y_{s},m_{s})$ and $Q(x_{t},y_{t},m_{t})$. Conventional supervised learning assumes P=Q (i.i.d). However, in real clinical scenarios, cross-center images often exhibit significant domain shifts, i.e., P≠Q.

### 2.1. ADAM-Net Model Overview

UDA technology aims to learn general features that are both discriminative and domain-invariant in the absence of target domain labels. This enhances the model’s cross-domain generalization ability [28]. Multi-task learning leverages a shared feature extractor to enable feature-level information sharing, thereby reducing the number of model parameters [26]. It also facilitates knowledge complementarity between tasks: segmentation provides gland morphological features that enhance classification decisions, while classification incorporates pathological knowledge that constrains the semantic plausibility of segmentation results. Based on these, we propose the ADAM-Net architecture shown in Figure 1, which consists of a shared feature extractor $G_{f}$, a segmentation decoder $G_{seg}$, a label predictor $G_{cls}$, and a domain discriminator $G_{dom}$. This framework aligns the feature distributions of the source and target domains through domain-adversarial training while jointly optimizing MG segmentation and MGD classification tasks. It improves classification accuracy and segmentation precision in the target domain, significantly enhancing cross-domain robustness and the potential for clinical deployment.

This paper builds the overall framework grounded in DANN, using ImageNet pre-trained ResNet18 [29] as $G_{f}$, with the Convolutional Block Attention Module (CBAM) [30] embedded into its shallow layers. By jointly employing channel and spatial attention modules, background interference is effectively suppressed and feature responses in lesion regions are enhanced, thereby improving feature representation capability. Each input sample *x* from $D_{s}$ and $D_{t}$ is passed through $G_{f}$ to obtain the feature representation $f\in R^{H\times W\times C}$, i.e., $f=G_{f}\left(x;\theta_{f}\right)$, where $\theta_{f}$ denotes the parameter vector of all layers in $G_{f}$. Feature *f* are then input into three submodules: $G_{f}$, $G_{cls}$, and $G_{dom}$. For the segmentation task, we design a U-Net–based decoder $G_{seg}$ [31]. Through a multi-level progressive upsampling strategy and skip connections, the spatial resolution is gradually restored, and high-dimensional semantic features from the encoder are fused. In addition, a CBAM attention module is integrated to enhance the response to target regions. The final output is the segmentation prediction map *m*. To further improve the model’s focus on the gland regions, we design an SGSA module. The predicted map *m* is passed through the convolutional transformation to generate a spatial attention heat map, which is then upsampled to match the size of *f* and multiplied element-wise with it, thereby explicitly enhancing the gland regions. Both $G_{cls}$ and $G_{dom}$ adopt a similar three-layer fully connected architecture, progressively reducing the dimensionality of the input feature $f^{\prime }$ to map it into the corresponding output space. $G_{cls}$ maps $f^{\prime }$ to the class label *y* for class prediction, while $G_{dom}$ maps $f^{\prime }$ to the domain label *d* for domain discrimination. To achieve domain invariance, a GRL is inserted between $G_{f}$ and $G_{dom}$, which performs weighted gradient reversal during backpropagation [25].

### 2.2. The Segmentation Decoder $G_{seg}$

**Figure 2:**
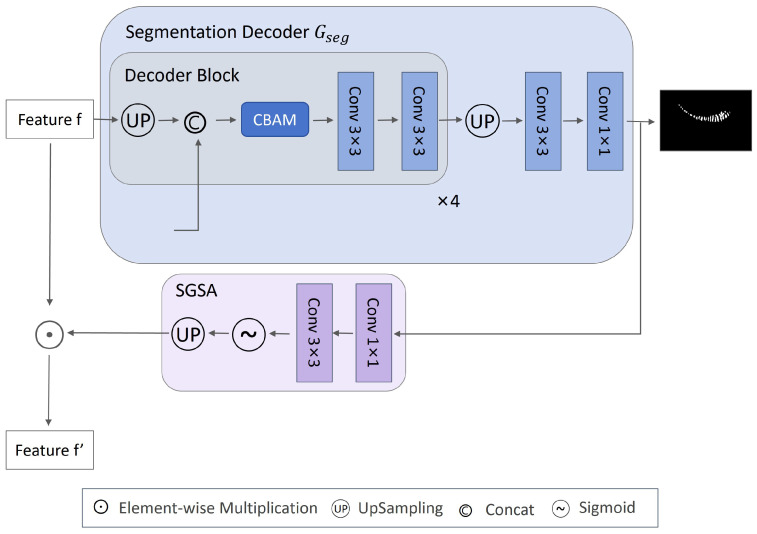
Architecture of the segmentation decoder $G_{seg}$ and the SGSA attention module, which collaboratively enhance structural feature extraction.

Based on the remarkable performance of U-Net in medical image segmentation, this paper designs a segmentation decoder built upon it, as shown in Figure 2. To progressively restore spatial resolution and fuse multi-scale features for improved localization accuracy and edge segmentation precision of the gland region, $G_{seg}$ is composed of four progressively upsampling decoding blocks and a final prediction layer, with its overall structure aligned to $G_{f}$ hierarchy. Each decoding block mainly consists of four components: first, transposed convolution is applied to *f* for 2× upsampling; then, skip connections are used to integrate the features of $G_{f}$ corresponding layer with the upsampled features, thereby supplementing spatial details; next, the CBAM attention mechanism is incorporated to enhance the model’s perception of key regions; finally, two 3×3 convolutional layers combined with batch normalization [32] and the ReLU activation function are applied for feature refinement. The channel configuration of each decoder stage decreases progressively with decoding depth: 512→256,256→128,128→64,64→64. The feature map output by the decoder is restored to the original resolution through bilinear interpolation and then mapped to the class dimension $K_{seg}$ ($K_{seg}=2$, representing background and foreground) through two convolutional layers, producing the segmentation prediction *m*, i.e., $m=G_{seg}\left(f;\theta_{seg}\right)$, where $\theta_{seg}$ denotes the parameters in $G_{seg}$. Since MG segmentation labels are available in $D_{s}$, the supervised learning strategy is adopted to train the network on $D_{s}$. The segmentation loss $L_{seg}$ is defined as:

(1) $$\min L_{seg}\left(\theta_{f},\theta_{seg}\right)=\sum_{i=1..n_{s}}L_{seg}\left(G_{seg}\left(G_{f}\left(x_{i}^{s};\theta_{f}\right);\theta_{seg}\right),m_{i}^{s};\omega =\left[0.4,1.0\right]\right)$$

where ω is the class weight, used to mitigate the imbalance between background and foreground classes. $L_{seg}$ is computed using a weighted cross-entropy loss function. During training, both $\theta_{f}$ and $\theta_{seg}$ are jointly optimized to minimize $L_{seg}$ on $D_{s}$.

### 2.3. SGSA Module

SGSA leverages the segmentation-predicted gland mask to generate a spatial attention heatmap that dynamically guides the feature responses of the classification branch and the domain discriminator. This forces them to focus on diagnostically meaningful gland regions while suppressing interference from background structures such as the eyeball and eyelashes. The SGSA module explicitly injects anatomical structure information into high-level semantic tasks, effectively enhancing the model’s interpretability and its ability to learn domain-invariant features. Specifically, the logit output from the segmentation head is denoted as $S\in R^{B\times K_{seg}\times H_{seg}\times W_{seg}}$. To compress its channel dimension and learn spatial saliency, *S* is first input into a lightweight convolutional module, consisting of a 3×3 convolution layer followed by batch normalization and a ReLU activation. This is then processed by a 1×1 convolution layer and a Sigmoid activation function to generate the normalized attention map $A\in {\left[0,1\right]}^{B\times 1\times H_{seg}\times W_{seg}}$, i.e., $A=\sigma ({Conv}_{1\times 1}\left(ReLU\left(BN\left({Conv}_{3\times 3}\left(S\right)\right)\right)\right))$, where σ(·) denotes the Sigmoid activation function. In order to align with the spatial dimensions of *f*, *A* is upsampled via bilinear interpolation to the same dimensions as *f*. Subsequently, element-wise multiplication is performed to obtain the enhanced feature map $f^{\prime }$, i.e., $f^{\prime }=f⊙UpSample\left(A\right)$. The structure of the SGSA module is illustrated in Figure 2. To further standardize its activation behavior, the attention consistency loss $L_{cons}$ is employed as a regularization term:

(2) $$\min L_{cons}\equiv \frac{1}{B}\sum_{i=1}^{B}\left(\frac{1}{HW}\sum_{h=1}^{H}\sum_{w=1}^{W}{\left(A_{i}\left(h,w\right)-\delta \right)}^{2}\right)$$

where δ denotes the predefined attention activation expectation value. $L_{cons}$ is computed using the mean squared error (MSE) loss function. During training, minimizing $L_{cons}$ enables the model to learn more focused and stable attention responses, effectively suppress background noise, and enhance the robustness of feature representations.

### 2.4. The Label Predictor $G_{cls}$

The original DANN’s $G_{cls}$ and $G_{dom}$ consist of stacked shallow fully connected layers. This structure has limited capability to represent the complex high-level semantic features in Meibography, thereby constraining the model’s discriminative performance. Therefore, in this study, $G_{cls}$ and $G_{dom}$ were reconstructed: while keeping the input feature dimensions (512×7×7) unchanged, the hidden layers’ dimension and depth were increased to enhance the model’s nonlinear mapping capability, resulting in a final structure of 512×7×7−2048−1024−num_classes. In the classification task, the number of classes is $K_{cls}=4$. The Dropout layer [33] with a probability of 0.35 is inserted between the first two fully connected layers to improve generalization.

The attention-enhanced feature $f^{\prime }$ is flattened and then input into a three-layer fully connected classifier for MGD grade prediction, producing *y*, i.e., $y\equiv G_{cls}\left(f^{\prime };\theta_{cls}\right)$, where $\theta_{cls}$ denotes the parameters of $G_{cls}$. Since Meiboscore labels are available in $D_{s}$, the supervised learning strategy is applied to optimize the classification task on $D_{s}$. The classification loss $L_{cls}$ is defined as:

(3) $$\min L_{cls}\left(\theta_{f},\theta_{cls}\right)=\sum_{i=1..n_{s}}L_{cls}\left(G_{cls}\left(G_{f}\left(x_{i}^{s};\theta_{f}\right)⊙UpSample\left(A\right);\theta_{cls}\right),y_{i}^{s}\right)$$

$L_{cls}$ is computed using the cross-entropy loss function. During training, both $\theta_{f}$ and $\theta_{cls}$ are jointly optimized to minimize $L_{cls}$ on $D_{s}$.

### 2.5. Adversarial Domain Adaptation Process

Since labels are unavailable in $D_{t}$, our objective is to effectively transfer the discriminative knowledge learned from $D_{s}$ to $D_{t}$. This requires the extracted features *f* to possess strong domain invariance, i.e., minimizing the distributional discrepancy between *P* and *Q* [34]. Therefore, we introduce DANN, where $G_{dom}$ distinguishes between source and target domain samples. Meanwhile, the GRL is inserted between $G_{f}$ and $G_{dom}$. During backpropagation, the GRL reverses the gradient direction, forcing $G_{f}$ to generate domain-invariant features that are difficult to discriminate, thereby confusing $G_{dom}$. This adversarial mechanism effectively reduces the distribution discrepancy between *P* and *Q*, thereby enhancing the model’s generalization ability in the target domain.

The structure of $G_{dom}$ is similar to $G_{cls}$, except that the final number of output classes is set to $K_{dom}=2$ (corresponding to source domain label 0 and target domain label 1). During training, $f^{\prime }$ is input into $G_{dom}$ to obtain the domain prediction *d*, i.e., $d=G_{dom}\left(f^{\prime };\theta_{dom}\right),d\in \left\{0,1\right\}$, where $\theta_{dom}$ denotes the parameter set of $G_{dom}$. $f^{\prime }$ is a high-dimensional dynamic feature whose distribution undergoes continuous changes during the training process, making it difficult for traditional static distribution measurement methods to effectively capture their differences. Therefore, in this work, the domain classification loss $L_{dom}$ is used to dynamically estimate the similarity between distributions of two domains. Specifically, to obtain domain-invariant features, we simultaneously optimize two adversarial objectives: on the one hand, updating $\theta_{f}$ to maximize $L_{dom}$, so that the feature distributions of two domains become as close as possible; on the other hand, updating $\theta_{dom}$ to minimize $L_{dom}$ [25,35]. This adversarial process is essentially a min-max optimization game [18], where adversarial training achieves a unification between domain discriminability and domain invariance [36]. $L_{dom}$ is expressed as:

(4) $$\max_{\theta_{f}}\min_{\theta_{dom}}L_{dom}\left(\theta_{f},\theta_{dom}\right)=\sum_{\begin{array}{c} i=1..n_{s}\\ j=1..n_{t} \end{array}}L_{dom}\left(G_{dom}\left(G_{f}\left(x_{i}^{s};x_{j}^{s};\theta_{f}\right)⊙UpSample\left(A\right);\theta_{dom}\right),d_{i}\right)$$

$L_{dom}$ is computed using the binary cross-entropy (BCE) loss function.

During forward propagation, the GRL functions as an identity transform, directly transmitting the input features. During backpropagation, it receives gradients from the subsequent layer and multiplies them by −λ before passing them to the preceding layer [25]. Here, λ is the gradient reversal weight and is not updated through backpropagation. A progressive training strategy is adopted, where λ monotonically increases from 0 to 1, following a sigmoid curve as training progresses, thereby gradually enhancing the model’s focus on the domain discrimination task. This strategy can be expressed as:

(5) $$\lambda_{p}\equiv \frac{2}{1+\exp(-\gamma \times p)}-1$$

where p∈[0,1] represents the training progress, and γ(>0) controls the steepness of the curve, which is set to 8 via a grid search.

### 2.6. Joint Loss Function $L_{total}$

To achieve the collaborative optimization of classification, segmentation, and domain adversarial tasks, the total objective loss function of the ADAM-Net model $L_{total}$ is obtained by combining Equations (1)–(5) as follows:

(6) $$L_{total}=\alpha L_{cls}\left(\theta_{f},\theta_{cls}\right)+\beta L_{seg}\left(\theta_{f},\theta_{seg}\right)-\lambda L_{dom}\left(\theta_{f},\theta_{dom}\right)+\mu L_{cons}$$

where the loss weight coefficient μ of $L_{cons}$ is determined via grid search and is finally set to μ=0.1. The loss weight coefficients α and β of $L_{cls}$ and $L_{seg}$ are dynamically adjusted through an uncertainty-weighted strategy between tasks to adaptively balance the loss magnitudes in multi-task learning [27]. The core idea of the uncertainty-weighted strategy is that the loss weight of each task is inversely proportional to its uncertainty (noise level). The weighting is computed as:

(7) $$L_{i}\equiv \frac{1}{2\sigma_{i}^{2}}L_{i}+\log\sigma_{i}^{2}$$

where $\sigma_{i}$ denotes the task-related noise level, characterizing the prediction uncertainty of the task, and is treated as a learnable parameter.

This loss fusion strategy effectively coordinates the weight relationships among multiple tasks within a unified optimization framework. It not only reinforces the model’s effective feature learning ability on $D_{s}$ but also significantly enhances cross-domain transfer ability and spatial awareness of key anatomical regions. It ultimately enables collaborative optimization and performance improvement across classification, segmentation, and domain adversarial tasks.

## 3. Experiments

This section presents the experimental analysis of the proposed ADAM-Net model. To validate the effectiveness of the proposed method, we first compare the performance of several mainstream UDA methods on the MGD classification task. Next, systematic ablation experiments are conducted to evaluate the contribution of each key module. In addition, visualization techniques are employed to analyze the model’s regional localization and feature alignment effects.

### 3.1. Datasets Description

The ADAM-Net model is evaluated on the open-source dataset MGD-1K and three real-world datasets. The MGD-1K dataset contains 1000 Meibography images with a resolution of 1280×640, each with precise MG and eyelid plate segmentation labels. Following the criteria proposed by Pflugfelder et al., the severity of MGD is categorized into four grades (Grade 0: no gland dropout; Grade 1: <33% gland dropout; Grade 2: 33–66% gland dropout; Grade 3: >66% gland dropout), with the specific class distribution shown in Table 2 [4]. All images were acquired using the LipiView II ocular surface interferometer (LV II; TearScience Inc., Morrisville, NC, USA) [37].

**Figure 3:**
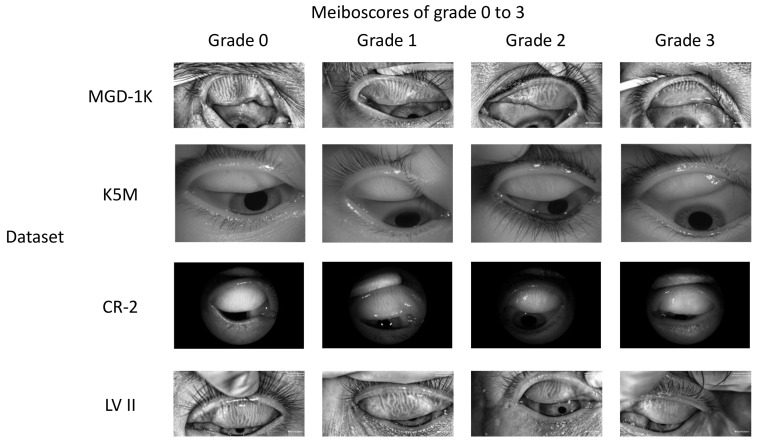
Representative Meibography images of Grade 0 to 3 from four datasets.

Real-world datasets were collected in clinical settings, where issues such as insufficient resolution, reflective occlusion, and incomplete eyelid eversion are commonly present [12]. These limitations significantly increase the difficulty of practical diagnosis and impose higher demands on the model’s robustness and generalization ability. Initially, data screening was performed, and the detailed statistics of each dataset after screening are shown in Table 2. Among them, the K5M and CR-2 datasets were collected from Zhuhai People’s Hospital (Zhuhai Hospital Affiliated to Jinan University). The K5M dataset was collected using the Keratograph 5M dry eye examination device (K5M; Oculus, Wetzlar, Germany) [38]. It contains 95 Meibography images with a resolution of 1360×1024. The CR-2 dataset was collected using the Canon CR-2 AF fundus camera (Canon; Canon, Japan) and contains 115 Meibography images with a resolution of 2736×1824. The LV II dataset was collected from Aier Eye Hospital using the LipiView II ocular surface interferometer and contains 264 Meibography images with a resolution of 1280×640. All real-world datasets contain only MGD classification labels and lack segmentation labels. The classification labels were precisely annotated by three annotators under the supervision of ophthalmologists, with the final results determined by a voting mechanism. Examples of different grades from the above datasets are shown in Figure 3. This study strictly follows the principles of the Declaration of Helsinki and was approved by the Ethics Committee of Zhuhai People’s Hospital (Zhuhai Hospital Affiliated to Jinan University) (Approval No.: [2024]-KT-67).

**Figure 4:**
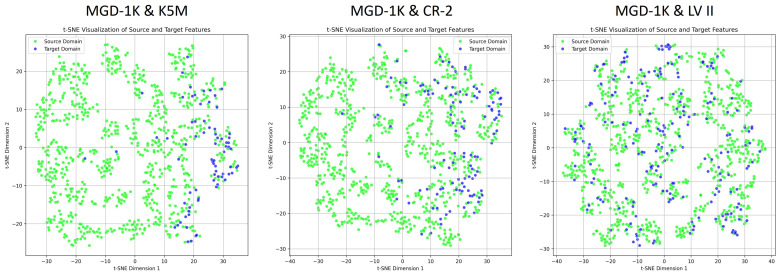
t-SNE visualization of feature distributions before domain adaptation across the source domain and three target domains.

Figure 4 illustrates the feature distribution differences across the source domain (MGD-1K) and the three target domains (K5M, CR-2, LV II). High-dimensional features are reduced to two dimensions for visualization using t-distributed Stochastic Neighbor Embedding (t-SNE), showing that all three target domains exhibit varying degrees of domain shift relative to the source domain. It is noteworthy that although the MGD-1K and LV II datasets were collected using the same equipment and have similar image styles, the MGD-1K dataset underwent strict screening and preprocessing, while the LV II dataset was collected directly in clinical settings. Consequently, the image quality of LV II is more susceptible to factors such as the physicians’ operation and the patients’ cooperation, resulting in greater variability. This discrepancy may lead to a reduction in the model’s generalization performance in real-world applications.

### 3.2. Evaluation Metrics

Classification: The confusion matrix systematically presents the model’s classification accuracy across different categories by comparing the ground truth labels with the predicted results [39]. Its diagonal elements represent the number of samples that have been correctly classified, while the off-diagonal elements reflect misclassification and the degree of confusion between different categories.

Accuracy is a metric for evaluating the overall classification performance of the model, representing the proportion of correctly classified samples to the total number of samples [40]:

(8) $$Accuracy\equiv \frac{\sum_{i=1}^{K}TP_{i}}{N}$$

where *K* is the total number of classes, $TP_{i}$ denotes the number of correctly classified samples in class *i*, and *N* is the total number of samples.

Precision is used to evaluate the proportion of samples predicted as positive that are actually positive, reflecting the reliability of the predictions [41]:

(9) $$Precision=\frac{\sum_{i=1}^{K}TP_{i}}{\sum_{i=1}^{K}\left(TP_{i}+FP_{i}\right)}$$

where $FP_{i}$ denotes the number of samples from class *i* that are incorrectly predicted as positive.

Recall measures the model’s ability to correctly identify positive samples, defined as the proportion of actual positive samples that are correctly predicted [41]:

(10) $$Recall=\frac{\sum_{i=1}^{K}TP_{i}}{\sum_{i=1}^{K}\left(TP_{i}+FN_{i}\right)}$$

where $FN_{i}$ denotes the number of samples from class *i* that are incorrectly predicted as negative.

The F1-score is the harmonic mean of precision and recall [41]:

(11) $$F1-score=2\times \frac{Presicion\times Recall}{Presicion+Recall}$$

The MCC-score further integrates true positives, true negatives, false positives, and false negatives [42]:

(12) $$MCC-score=\frac{\sum_{i=1}^{K}\left(TP_{i}\times TN_{i}-FP_{i}\times FN_{i}\right)}{\sqrt{\sum_{i=1}^{K}\left(TP_{i}+FP_{i}\right)\left(TP_{i}+FN_{i}\right)\left(TN_{i}+FP_{i}\right)\left(TN_{i}+FN_{i}\right)}}$$

where $TN_{i}$ denotes the number of samples in class *i* that are correctly predicted as negative. The MCC-score ranges from [−1,1], with higher values indicating more balanced and reliable classification performance.

The F1-score and MCC-score are both used to evaluate the performance of classification models. They are particularly valuable for datasets with class imbalance.

Segmentation: The Dice coefficient is commonly used to evaluate the overlap between binary segmentation results and ground truth labels [43]:

(13) $$Dice=\frac{2\times |A\cap B|}{\left|A|+|B\right|}$$

where *A* denotes the segmentation prediction, *B* denotes the ground truth, |A∩B| is the number of pixels in their intersection, and |A|+|B| is the total number of pixels in both sets.

IoU is another commonly used segmentation metric, defined as the ratio of the intersection over the union between the predicted region and the ground truth:

(14) $$IoU=\frac{\left|A\cap B\right|}{\left|A\cup B\right|}$$

where |A∪B| denotes the number of pixels in the union of the two regions.

Although both Dice and IoU are used to measure the overlap between predictions and ground truth, their calculation methods differ: typically, Dice scores are slightly higher than IoU scores. Dice is more sensitive to region overlap, making it suitable for medical images with small foregrounds or class imbalance. IoU is more stringent and imposes a stronger penalty on misclassified pixels. In practice, the two metrics are often combined to comprehensively evaluate the model’s segmentation performance.

### 3.3. Experimental Setup

#### 3.3.1. Implementation Details

The experiments used the publicly available MGD-1K dataset as the source domain, with three real-world datasets (K5M, CR-2, LV II) serving as target domains in sequence. To avoid the risk of data leakage, this study strictly followed an anatomical region-level isolation principle during data partitioning. Specifically, all images of the same eyelid region from the same patient (e.g., left upper eyelid, left lower eyelid) belong exclusively to one subset of the training, validation, or test set. This ensures that there is no image- or structural-level overlap among different subsets, thereby effectively preventing the model from memorizing individual or localized anatomical features and avoiding overfitting. A stratified sampling strategy was employed in the specific partitioning to maintain consistent category proportions. In the MGD-1K dataset, each class was split into training, validation, and test sets at a ratio of 7:1:2, and the real-world datasets were divided at a ratio of 6:1:3. This ensures that test sets accurately represent the real clinical data distribution [6]. Due to severe class imbalance in the dataset, insufficient samples in minority classes can easily cause the model to overfit the majority classes. To address this issue, minority classes were oversampled and augmented only during training, using operations such as random flipping, rotation, cropping, Gaussian noise injection, and brightness perturbation to balance the class distribution [12].

This study established an experimental environment on a computing platform equipped with an NVIDIA GeForce RTX 3090 GPU. The proposed ADAM-Net model was implemented using the PyTorch 2.7.0 deep learning framework (CUDA 11.8 support) and Python 3.10. The model employs a pretrained ResNet-18 on ImageNet as its feature extractor backbone network. Only source domain labels were used during training. The input images were uniformly resized to 224×224 pixels. The Adam optimizer with a learning rate of $1\times 10^{-4}$ was employed, with a batch size of 16, for end-to-end training over 200 epochs. The forward computation process yields prediction results, which are subsequently optimized by combining multiple losses. Backpropagation updates the network parameters. The model’s hyperparameters are tuned on the validation set. The test set remains strictly independent and sealed throughout training and tuning process, and is used only for final performance evaluation. After determining the optimal configuration, the training and validation sets are merged to retrain the model and obtain the final weights. This approach makes the most of limited samples while effectively preventing overfitting and information leakage in the test set. During the testing phase, the test images from both the source and target domains were input into the final model to obtain class labels and segmentation masks, thereby evaluating the model’s cross-domain generalization ability.

#### 3.3.2. Parameter Tuning Strategy

**Figure 5:**
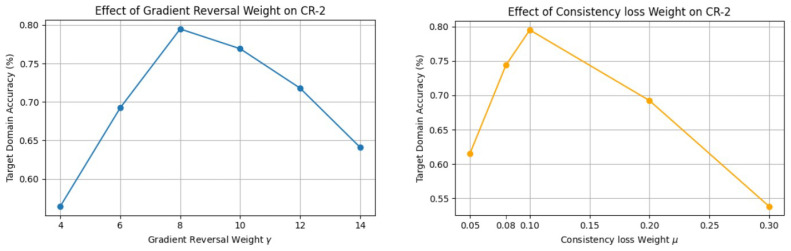
Parameter sensitivity analysis of ADAM-Net with respect to γ and μ on the cross-domain task MGD-1K→CR-2.

This study focuses on demonstrating the tuning process of the key hyperparameters γ and μ. Due to the pronounced differences in domain shift across target domains, the more challenging MGD-1K→CR-2 task, which exhibits a larger domain discrepancy, is selected as the primary scenario for hyperparameter tuning to improve the representativeness and stability of parameter selection. This setting exhibits large performance fluctuations in preliminary experiments, imposing higher demands on cross-domain generalization and therefore serving as a representative case. While keeping the rest of the training configuration fixed, perform a grid search on γ and μ separately. γ was selected from {4,6,8,10,12,14}, and μ was selected from {0.05,0.08,0.1,0.2,0.3}. Tuning was performed using the classification accuracy on the target-domain validation set as the primary metric, to evaluate the transfer performance of the model under different parameter configurations. As shown in Figure 5, as γ increases, the model’s performance on CR-2 first improves and then degrades. When γ is too small, the domain alignment constraint is insufficient to effectively reduce the domain discrepancy. When γ is too large, excessively strong adversarial constraint weakens feature discriminability, leading to performance degradation. The best performance on the target domain is achieved at γ=8, which is therefore adopted as the final setting. Similarly, when μ is too small, the structural constraint imposed by the multi-task framework is limited; when μ is too large, it may have an excessive regularization that adversely affects the classification task. The best trade-off on the target domain is achieved at μ=0.1, which is therefore used in all subsequent experiments. Although the tuning was primarily conducted on the MGD-1K→CR-2 task, applying the obtained parameters to the remaining cross-domain tasks (MGD-1K→K5M and MGD-1K→LV II) yielded stable and consistent performance improvements. This demonstrates that the selected hyperparameters exhibit strong cross-task generalization capabilities.

### 3.4. Experimental Results

#### 3.4.1. Comparison of Domain Adaptation Methods

To enhance the model’s adaptability to distributional differences in multi-center medical data, it is necessary to strengthen its cross-domain transferability. To validate the effectiveness and advancement of the ADAM-Net, this study compares it with current mainstream UDA methods in the cross-domain classification task of Meibography. The selected comparison methods include: DANN [25], Conditional Domain Adaptation Network (CDAN) [36], Deep Adaptation Network (DAN) [44], Joint Adaptation Network (JAN) [45], Margin Disparity Discrepancy for Unsupervised Domain Adaptation (MDD) [46], Batch Spectral Penalization for Adversarial Domain Adaptation (BSP) [47], and Minimum Class Confusion for Versatile Domain Adaptation (MCC) [48]. These methods cover various domain adaptation strategies such as adversarial learning, distribution matching, and feature constraints. All the above methods were executed under a unified experimental setup to ensure objective and comparable results.

The experimental results are shown in Table 3. We comprehensively evaluated the performance of different methods on cross-domain classification tasks using multiple evaluation metrics. The DANN and CDAN methods based on adversarial learning demonstrate significantly higher classification performance on the target domains compared to other methods, exhibiting stronger feature alignment and cross-domain generalization capabilities. It is worth noting that CDAN achieved a relatively high classification accuracy on the source domain in the MGD-1K→K5M task (84.58%), indicating that it can learn effective discriminative patterns from sufficiently annotated source domain data. However, its performance on the target domain drops markedly. This may be attributed to CDAN’s excessive reliance on domain-specific features in the source domain that are not directly related to pathological semantics, thereby limiting its cross-domain generalization capability. Further analysis of metrics such as precision, recall, and F1-score reveals that CDAN still exhibits certain limitations in its classification capabilities for a small number of categories. In contrast, distribution-matching methods (e.g., DAN, JAN, and MDD) and feature-regularization methods (e.g., BSP and MCC) show relatively limited performance in cross-domain Meibography classification tasks, with generally lower classification metrics. This may be attributed to the semantic complexity of medical images and the structural differences across domains, whereas adversarial learning mechanisms are more capable, in this context, to learn domain-invariant representations that remain both discriminative and robust across different domains. The proposed ADAM-Net demonstrates significant and stable advantages across all cross-domain tasks. In the MGD-1K→{K5M, CR-2, LV II}tasks, ADAM-Net achieves target domain classification accuracies of 75.86%, 74.29%, and 81.01%, respectively. It also significantly outperforms existing methods in key metrics including Precision, Recall, F1-score, and MCC-score. The notably superior MCC-score further indicates that the model maintains stable classification performance and high prediction consistency even under class imbalanced conditions. The results demonstrate that ADAM-Net attains more effective cross-domain feature alignment when confronted with complex domain discrepancies and class imbalances. It is also capable of extracting discriminative and robust domain-invariance features, thereby demonstrating exceptional cross-domain generalization capabilities.

#### 3.4.2. Ablation Study

To systematically evaluate the specific contributions of the domain adaptation mechanism, multi-task learning, and attention modules, we first construct a ‘source-only’ model based on ResNet18 as the Baseline. This model does not incorporate any additional modules and undergoes supervised training solely on the MGD-1K to perform the MGD classification task. After training, the Baseline is directly applied to three unseen real-world datasets (K5M, CR-2, and LV II) for independent evaluation, in order to assess the generalization ability when relying solely on source-domain discriminative features.As shown in Table 4, the Baseline achieves an accuracy of 85.57% on the source domain, indicating strong intra-domain discriminative capability. However, its classification performance drops severely on the target domains, with accuracies of only 18.95% and 19.13% on K5M and CR-2, respectively, and 51.14% on LV II. It should also be noted that the Baseline achieves negative MCC-score on K5M and CR-2 (−0.1648 and −0.1259, respectively), indicating that its predictive performance on the target domains is even worse than random guessing. This result clearly reveals the limitations of conventional supervised learning in cross-domain scenarios and further validates the necessity of introducing domain adaptation mechanisms.

The ADAM-Net model is built upon the DANN architecture. Although the DANN has already demonstrated improved classification accuracy in the target domain compared to the Baseline, as shown in Table 4, its performance is still insufficient to meet the requirements of practical clinical applications. Analysis reveals that the original DANN’s $G_{cls}$ and $G_{dom}$ consist of stacked shallow fully connected layers. In this study, $G_{cls}$ and $G_{dom}$ were reconstructed: the hidden layers’ dimension and depth were increased to enhance the model’s nonlinear mapping capability, resulting in a final structure of 512×7×7−2048−1024−num_classes. To quantify the impact of this improvement on cross-domain performance, under the same training configuration, we compared the original DANN with the improved DANN (referred to as DANN-Head) in terms of classification metrics on the cross-domain tasks MGD-1K→{K5M, CR-2, LV II}. As shown in Table 4, DANN-Head achieved improved classification accuracy across all three target domains: K5M increased by 6.89% to 55.17%, CR-2 increased by 11.43% to 54.29%, and LV II increased by 2.52% to 58.22%. In addition to accuracy, DANN-Head also exhibits a consistent improvement trend in metrics such as F1-score and MCC-score. These results validate the effectiveness of enhancing the model’s nonlinear mapping capability.

Considering that the MGD classification task is highly dependent on the spatial structural features of gland morphology, the MG segmentation task can provide critical anatomical guidance for classification. The multi-task learning strategy enables the collaborative optimization of MGD classification and MG segmentation tasks, allowing the shared feature extractor to focus more effectively on the MG regions. Therefore, based on DANN-Head, a segmentation decoder was introduced to construct the DANN-Head+Seg multi-task framework to evaluate the auxiliary effect of the segmentation task on the classification task. Although the real-world target datasets lack segmentation annotations, preventing direct evaluation of segmentation quality, their spatial guidance effect can be indirectly verified through the improvement in classification accuracy. The loss weights between the segmentation and classification tasks are adaptively determined using the task uncertainty weighting strategy [27]. As shown in Table 4, in the cross-domain tasks MGD-1K→{K5M, CR-2, LV II}, DANN-Head+Seg achieved further improvements in classification accuracy over DANN-Head across all three target domains: K5M increased by 3.45% to 58.62%, CR-2 increased by 2.85% to 57.14%, and LV II increased by 5.07% to 63.29%. DANN-Head+Seg also shows concurrent improvements on F1-score and MCC-score, which are more suitable for evaluation under class-imbalanced conditions. These results validate the enhancing effect of the segmentation task on the classification task through anatomical priors.

**Figure 6:**
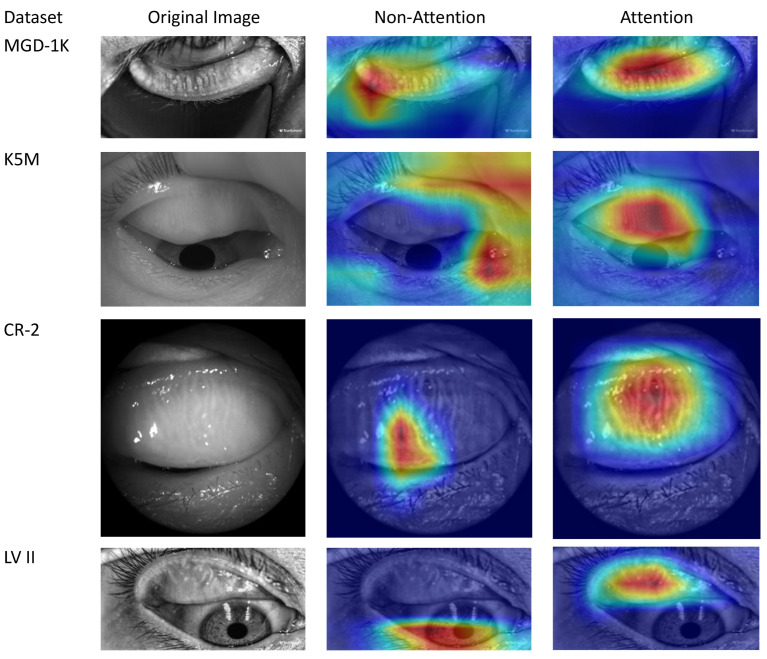
Grad-CAM visualization of activation regions with versus without attention mechanism across four datasets. Warmer colors (red) indicate regions with higher contribution to the classification decision, while cooler colors (blue) indicate lower contribution.

MG in Meibography images usually occupies only a local region, with grayscale characteristics similar to the background. Additionally, it is easily disturbed by complex structures such as eyelashes and the eyeball, resulting in insufficient model attention to the target region. As shown in Figure 6, the Gradient-weighted Class Activation Mapping (Grad-CAM) attention heatmaps [49] indicate that the DANN-Head+Seg model without the attention mechanism exhibits dispersed activation regions, making it difficult to focus on the MG area. This limits the model’s feature representation ability and cross-domain generalization performance. To address this issue, this study introduces the CBAM, embedding it into the shallow layers of the feature extractor and the segmentation decoder block to construct the DANN-Head+Seg+CBAM model. As shown in Table 4, in the cross-domain tasks MGD-1K→{K5M, CR-2, LV II}, the DANN-Head+Seg+CBAM model achieves consistent improvements in classification accuracy across all three target domains: an increase of 6.90% to 65.52% on K5M, 2.86% to 60.00% on CR-2, and 5.06% to 68.35% on LV II. In addition, DANN-Head+Seg+CBAM exhibits stable and consistent performance improvements across multiple other evaluation metrics.

**Figure 7:**
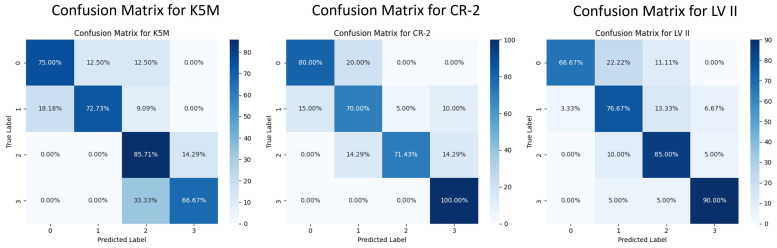
Classification confusion matrix of ADAM-Net model on three target domains.

**Figure 8:**
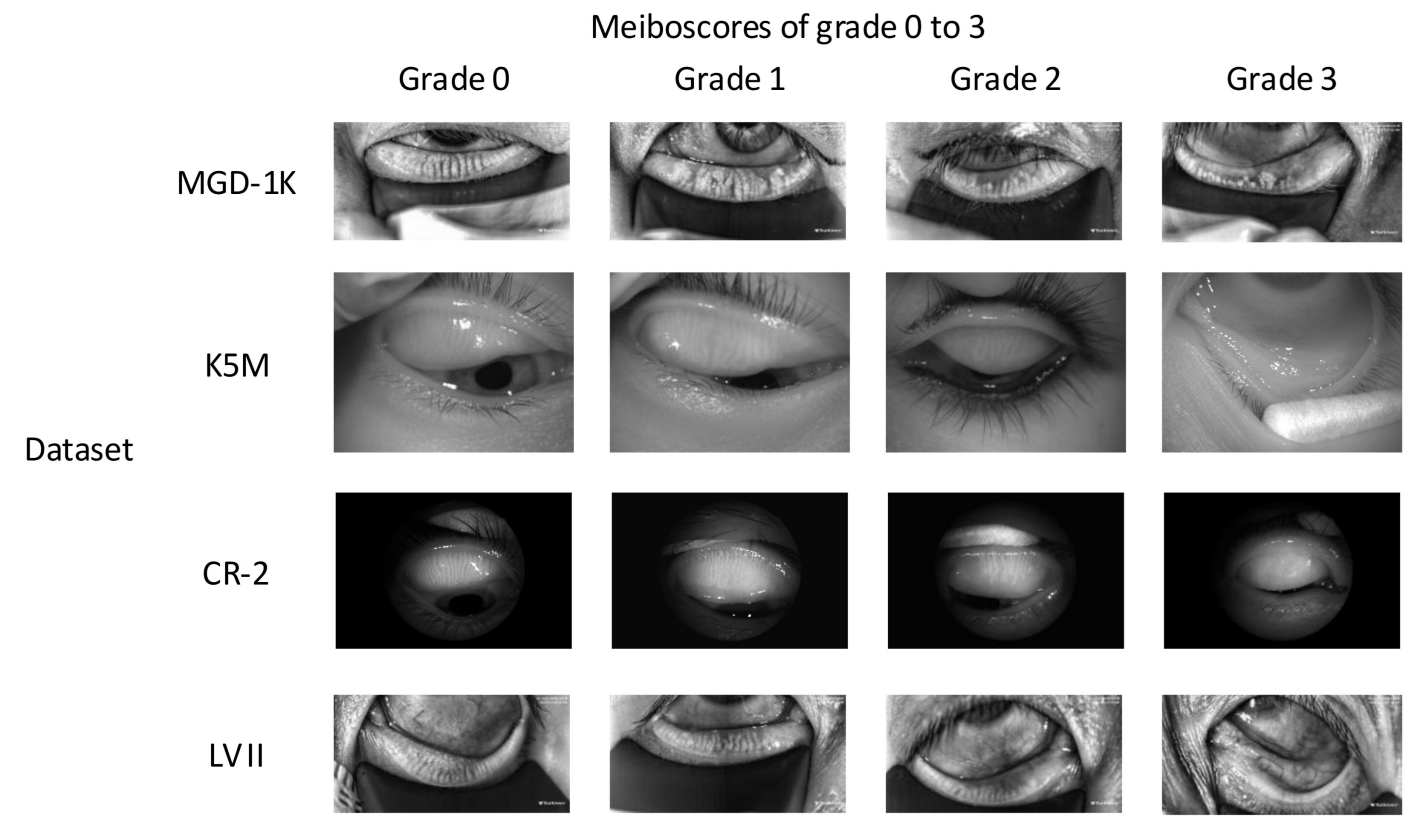
Representative correctly classified Meibography images of Grade 0 to 3 from four datasets using the proposed ADAM-Net.

Building upon the DANN-Head+Seg+CBAM model, the SGSA module is introduced to construct the final model, ADAM-Net (DANN-Head+Seg+CBAM+SGSA). As shown in Figure 6, the Grad-CAM visualization results demonstrate that with the combined effects of CBAM and SGSA, the model exhibits a more concentrated response to the MG region. It effectively suppresses background interference and enhances the perception of critical lesion areas. As shown in Table 4, ADAM-Net achieves significant overall performance advantages across all three cross-domain tasks: MGD-1K→{K5M, CR-2, LV II}. Compared with DANN-Head+Seg+CBAM, the classification accuracy on the target domains is further improved by 10.34% to 75.86%, 14.29% to 74.29%, and 12.66% to 81.01%, respectively, thereby outperforming the aforementioned comparison methods. This demonstrates the excellent cross-domain generalization capability of ADAM-Net under multi-center data settings. The F1-scores on the three target domains reach 74.17%, 72.01%, and 80.30%, respectively, indicating a good balance between precision and recall, with improved robustness in recognizing minority classes. And the MCC-scores are 0.6695, 0.6240, and 0.7357. As shown in Figure 7, the classification confusion matrices of the three target domains further demonstrate that the model not only achieves superior overall classification performance but also exhibits more balanced recognition across different atrophy grades, indicating strong inter-class discriminative capability. Examples of images correctly classified by ADAM-Net are shown in Figure 8.

**Figure 9:**
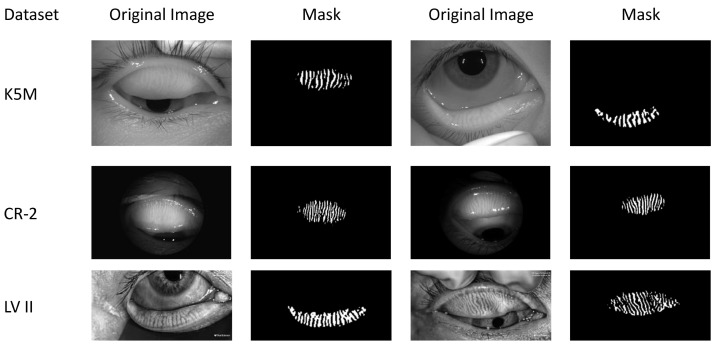
Examples of original images and corresponding gland segmentation masks from three target domains.

In the segmentation task, the model achieved a performance of Dice: 83.28%, and IoU: 71.35% on the source domain MGD-1K, indicating its capability to effectively segment MG regions. For the target-domain data, due to the lack of corresponding segmentation annotations, quantitative evaluation of the segmentation results is not feasible. Therefore, only qualitative analysis of the model’s segmentation behavior is conducted through visualization. As shown in Figure 9, the segmentation results on the target domain exhibit good consistency with the anatomical structure of the MGs in terms of overall morphology and spatial distribution. This suggests that the model can capture some spatial prior information related to the MG region under unsupervised conditions. It should be noted that these visualizations are provided only as auxiliary analysis to illustrate the model’s potential structural awareness, rather than as quantitative evidence of segmentation performance on the target domain.

#### 3.4.3. Statistical Analysis Across Multiple Seeds

To verify the robustness of ADAM-Net under different random initialization conditions and reduce the randomness bias of single-run results, this study repeated the experiments using five different random seeds: 42, 7, 123, 999, and 2023. Experimental variations under different random seeds are limited to the stratified partitioning of anatomical units, random initialization of model parameters, and data loading order, while all other experimental settings are strictly kept identical. Final results are reported in the form of mean ± standard deviation (mean ± std). Table 5 summarizes the multi-metric results of ADAM-Net on the three cross-domain tasks: MGD-1K→{K5M, CR-2, LV II}. The experimental results show that some metrics exhibit relatively large standard deviations in certain tasks, mainly due to the following reason: first, the target-domain sample size is limited, with a small number of anatomy-level units, making the evaluation results more sensitive to variations introduced by different random seeds in data partitioning and model initialization [50]. Second, the datasets exhibit significant class imbalance. Under different initialization conditions, the model’s ability to learn features of minority classes may fluctuate, which is more prominently reflected in class-distribution-sensitive metrics such as recall and the MCC-score [42]. Nevertheless, ADAM-Net still demonstrates good training robustness and result consistency under different random seeds.

#### 3.4.4. Visualization

To validate the regulatory effect of the attention mechanism on the model’s focus regions, this study employs the Grad-CAM method to visualize the model’s focus regions before and after the introduction of the attention mechanism. Grad-CAM generates class-specific heatmaps by leveraging the gradient information of target classes, highlighting the key regions the model focuses on during decision-making. These heatmaps are then superimposed on the original image to intuitively display the regions the model relies on when making predictions [49]. As shown in Figure 6, in the DANN-Head+Seg model without attention mechanisms, the model is susceptible to background interference, resulting in dispersed activation regions that often cover non-essential regions or miss lesion regions. With the incorporation of CBAM and SGSA attention mechanisms, ADAM-Net demonstrates a marked enhancement in its focus on lesion regions, with stronger responses and significantly reduced background interference. This indicates that the attention mechanisms effectively enhance the model’s ability to perceive critical semantic regions. Moreover, this result validates the pivotal function of attention mechanisms in enhancing complex medical image understanding and cross-domain diagnosis.

**Figure 10:**
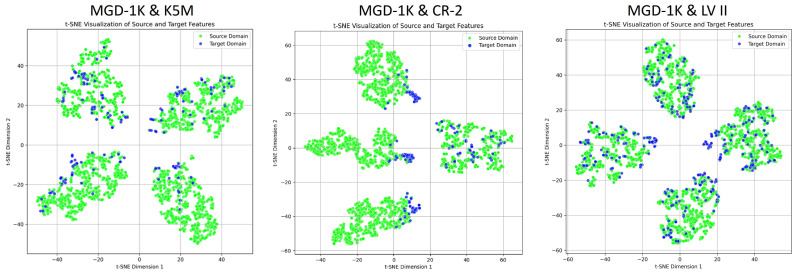
t-SNE visualization of feature distributions after domain adaptation with ADAM-Net across the source domain and three target domains.

To intuitively evaluate the effectiveness of the model in feature alignment, this study employs t-SNE to visualize the distribution of source and target domain data in the feature space. t-SNE can reduce high-dimensional features while preserving the local structural relationships between samples as much as possible [51]. This allows for the visualization of clustering tendencies and distribution differences among features from different domains or categories, thereby providing visual evidence for feature extraction and alignment effectiveness. Figure 4 shows the original feature distributions of the source and target domains, where a clear domain shift can be observed, and the class clustering structures appear loose. In contrast, Figure 10 illustrates that after incorporating domain-adversarial training, multi-task learning strategies, and attention mechanisms, ADAM-Net achieves significantly overlapping feature distributions between the source and target domains. The inter-domain boundaries become blurred, and the intra-class structures appear compact, demonstrating excellent feature alignment and discriminative capability. This result validates the effectiveness of the proposed method in mitigating cross-domain distribution shifts.

### 3.5. Failure Cases Analysis

**Figure 11:**
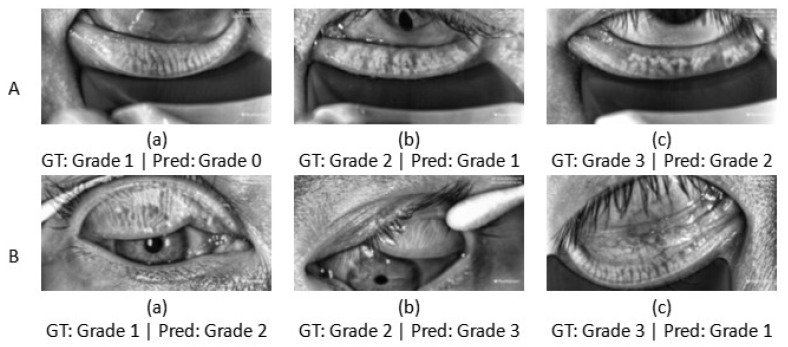
Representative misclassified examples of the proposed ADAM-Net. Ground-truth (GT) grades and predicted (Pred) grades are shown below each image. (**A**) Borderline samples located near grade boundaries. (**B**) Meibography images affected by real-world noise, including specular (**a**) reflections, (**b**) cotton swabs, and (**c**) eyelashes.

Although ADAM-Net exhibits overall stable and superior classification performance across the three cross-domain tasks, some samples were misclassified. To further analyze the limitations of the proposed model and improve its clinical interpretability, this study conducts an analysis of misclassified samples. The failure cases are mainly concentrated in the following three categories: (1) Figure 3 shows that the significant domain shift exists between the source and target domains. Although this study introduced a DA strategy, the method itself has limitations in generalization [28]. This makes it difficult to completely eliminate differences in feature distributions under extreme shifts. This results in an inability to accurately classify some target domain samples. (2) The MGD grading criteria adopted in this study define explicit quantitative boundaries (e.g., Grade 1 and Grade 2 are separated by a 33% gland loss threshold). As shown in Figure 11A, samples located near these boundaries exhibit morphological characteristics that are highly similar to those of adjacent grades [6]. Even for human experts, determining the final grade often requires multiple rounds of annotation and a voting mechanism. Consequently, the model is prone to classification uncertainty in such borderline cases. (3) As Figure 11B shows, Meibography images often contain background noise such as specular reflections, cotton swabs, and eyelashes. This noise obscures local structural features of the meibomian glands and interacts with cross-domain imaging variations, creating a compounding effect that further complicates the model’s ability to extract and distinguish effective features.

## 4. Conclusions

This study proposes an attention-guided unsupervised domain adaptation multi-task network, ADAM-Net, to address the challenges in automated MGD diagnosis, including cross-center domain shift, the isolation of classification and segmentation tasks, and background noise interference. The study demonstrates that ADAM-Net can significantly enhance model generalization without relying on target domain annotations, achieving collaborative optimization of both MG segmentation and MGD classification tasks. Specifically, this method constructs a domain-adversarial learning mechanism through GRL to effectively align the feature distributions of the source and target domains. By jointly optimizing the classification and segmentation tasks, it achieves dual-task knowledge transfer, thereby improving classification performance on unlabeled target domains and enhancing cross-domain generalization capability. Meanwhile, SGSA generates attention maps based on segmentation masks and incorporates consistency loss for regularization, enhancing the model’s perception of ROIs. The proposed method was validated across multiple cross-center MGD datasets. The experimental results demonstrate that ADAM-Net achieves final average classification accuracies of 77.93%, 74.86%, and 81.77% on the K5M, CR-2, and LV II target domains, respectively, significantly outperforming existing mainstream UDA methods. Additionally, the model demonstrates strong performance across multiple metrics, including the F1-score and MCC-score, indicating its robustness in class imbalance scenarios. The segmentation task was mainly evaluated through visualization of the segmentation masks and analysis of structural consistency. The results indicate that the model can still generate structurally plausible and clinically interpretable segmentation outcomes under cross-domain settings. Overall, ADAM-Net explores a tripartite synergy of domain adaptation, multi-task learning, and anatomy-guided approaches in MGD diagnosis, providing a reusable technical framework for cross-center applications in medical imaging.

It should be noted that although this study has made progress in improving the classification performance of the target domain and assisting segmentation discrimination, certain limitations remain when dealing with unsupervised cross-domain tasks involving significant distribution discrepancies. First, due to the lack of pixel-level segmentation annotations in the target domain, the performance improvement in the segmentation branch on the target domain in this study is mainly evaluated through qualitative visual analysis, and comprehensive quantitative metrics cannot yet be provided. Second, the absence of target-domain labels limits the model’s ability to fully learn discriminative features, making it susceptible to unstructured noise and domain bias. Moreover, adversarial training or statistical alignment methods struggle to achieve sufficient semantic-level alignment in scenarios with large domain discrepancies. Future research may further explore self-supervised learning strategies based on the target domain to fully uncover its latent semantic structures and spatial patterns. This would enhance the model’s feature representation and cross-domain generalization capabilities, thereby improving the deployability of intelligent medical image diagnostic systems in real clinical settings.

## Figures and Tables

**Table 1:** Overview of existing Meibography analysis methods and the capabilities of the proposed ADAM-Net. Symbols: ✓ explicit capability, △ implicit or indirect capability, × not supported. The bold refers to the method proposed in this paper.

Method	Unsupervised Domain Adaptation	Joint MG Segmentation & MGD Classification	Attention Mechanism	ROI-Aware/Anatomy-Guided
Source-only Classifier	×	×	×	×
Source-only Segmenter	×	×	×	×
Mainstream UDA methods	✓	×	×	×
DANN-Head	✓	×	×	×
DANN-Head+Seg	✓	✓	×	△
DANN-Head+Seg+CBAM	✓	✓	✓	△
**ADAM-Net**	✓	✓	✓	✓

**Table 2:** Summary of four Meibography datasets for meibomian gland dysfunction diagnosis, detailing imaging devices and image distributions across clinical grades (Grade 0 to 3).

Datasets	Devices	Total	Grade 0	Grade 1	Grade 2	Grade 3
MGD-1K	LipiView II Ocular Surface	1000	180	612	168	40
K5M	Keratograph 5M	95	25	38	22	10
CR-2	CR-2 AF	115	15	68	22	10
LV II	LipiView II Ocular Surface	264	30	100	66	68

**Table 3:** Comparison of classification performance metrics across domain adaptation methods on cross-domain tasks. Note: upper row represents source domain result, lower row represents target domain result. The bold indicates the best performance among different methods under identical tasks and experimental settings.

Task	Metrics	DANN	CDAN	DAN	JAN	MDD	BSP	MCC	ADAM-Net
MGD-1K→K5M	Accuracy	0.7662 0.4828	**0.8458** 0.4138	0.7811 0.3448	0.7512 0.3448	0.7512 0.3793	0.6866 0.3793	0.6915 0.3103	0.8358 **0.7586**
	Precision	0.6491 0.4874	0.7292 0.3828	0.6544 0.3263	0.6302 0.3056	0.6273 0.3750	0.5578 0.3618	0.5594 0.3173	**0.7899** **0.7431**
	Recall	0.7589 0.5124	0.8172 0.3849	0.7357 0.3394	0.7129 0.2788	0.7129 0.3707	0.6331 0.4012	0.6351 0.3688	**0.8427** **0.7503**
	F1-score	0.6850 0.4886	0.7604 0.3774	0.6823 0.3277	0.6585 0.2887	0.6568 0.3600	0.5795 0.3500	0.5824 0.3186	**0.8102** **0.7417**
	MCC-score	0.6242 0.2864	**0.7443** 0.1904	0.6387 0.1033	0.5905 0.0945	0.5925 0.1711	0.5026 0.1492	0.5079 0.0738	0.7288 **0.6695**
MGD-1K→CR-2	Accuracy	0.7512 0.4286	0.7363 0.4857	0.7214 0.4000	0.7363 0.3714	0.7463 0.4286	0.6667 0.3714	0.7015 0.3429	**0.7910** **0.7429**
	Precision	0.6368 0.3860	0.6607 0.4469	0.6876 0.3750	0.6767 0.3274	0.6263 0.4250	0.6119 0.3396	0.5910 0.3389	**0.7421** **0.6949**
	Recall	0.7532 0.4262	0.7418 0.5363	0.7463 0.4048	0.7418 0.3548	0.6994 0.4738	0.6901 0.3780	0.6778 0.4131	**0.8138** **0.8036**
	F1-score	0.6720 0.3792	0.6914 0.4662	0.7046 0.3672	0.7017 0.3186	0.6478 0.3985	0.6326 0.3336	0.6168 0.3403	**0.7692** **0.7201**
	MCC-score	0.6070 0.2161	0.5812 0.2695	0.5656 0.1504	0.5762 0.1197	0.5810 0.2447	0.4648 0.1329	0.5197 0.1084	**0.6604** **0.6240**
MGD-1K→LV II	Accuracy	0.8358 0.5570	0.8458 0.5190	**0.8507** 0.5063	0.8159 0.5190	0.8010 0.5316	0.7910 0.4810	0.7662 0.4557	0.8408 **0.8101**
	Precision	0.7216 0.5437	0.7368 0.5125	0.7605 0.4824	0.7387 0.4914	0.6847 0.5596	0.6815 0.4640	0.6522 0.4298	**0.8136** **0.8187**
	Recall	0.8276 0.5556	0.8172 0.5417	0.8738 0.4944	0.8293 0.5139	0.7985 0.5431	0.7842 0.4778	0.7486 0.4417	**0.8895** **0.7958**
	F1-score	0.7544 0.5464	0.7675 0.5129	0.8027 0.4838	0.7732 0.4896	0.7237 0.5272	0.7197 0.4677	0.6850 0.4336	**0.8448** **0.8030**
	MCC-score	0.7286 0.3881	0.7407 0.3529	**0.7605** 0.3218	0.6966 0.3468	0.6836 0.3856	0.6642 0.2884	0.6157 0.2514	0.7445 **0.7357**

**Table 4:** Ablation study on classification performance metrics of different models on cross-domain tasks. Note: upper row represents source domain result, lower row represents target domain result. The bold indicates the best-performing ablation configuration under identical tasks and experimental settings.

Task	Metrics	Baseline	DANN	DANN-Head	DANN-Head +Seg	DANN-Head +Seg+CBAM	ADAM-Net
MGD-1K→K5M	Accuracy	**0.8557** 0.1895	0.7662 0.4828	0.7811 0.5517	0.7960 0.5862	0.8060 0.6552	0.8358 **0.7586**
	Precision	0.7814 0.1702	0.6491 0.4874	0.7163 0.5762	0.7276 0.6081	0.7203 0.6470	**0.7899** **0.7431**
	Recall	**0.8899** 0.1649	0.7589 0.5124	0.7756 0.5709	0.7764 0.6542	0.8058 0.7167	0.8427 **0.7503**
	F1-score	**0.8246** 0.1658	0.6850 0.4886	0.7415 0.5676	0.7493 0.6226	0.7505 0.6679	0.8102 **0.7417**
	MCC-score	**0.7633** −0.1648	0.6242 0.2864	0.6381 0.3833	0.6559 0.4340	0.6940 0.5313	0.7288 **0.6695**
MGD-1K→CR-2	Accuracy	**0.8557** 0.1913	0.7512 0.4286	0.7363 0.5429	0.7015 0.5714	0.7612 0.6000	0.7910 **0.7429**
	Precision	**0.7814** 0.1706	0.6368 0.3860	0.6348 0.5028	0.7291 0.5199	0.7791 0.5325	0.7421 **0.6949**
	Recall	**0.8899** 0.1962	0.7532 0.4262	0.7471 0.5845	0.7363 0.5970	0.8054 0.6095	0.8138 **0.8036**
	F1-score	**0.8246** 0.1578	0.6720 0.3792	0.6735 0.5134	0.7195 0.5321	0.7810 0.5515	0.7692 **0.7201**
	MCC-score	**0.7633** −0.1259	0.6070 0.2161	0.5830 0.3708	0.5249 0.3996	0.6106 0.4247	0.6604 **0.6240**
MGD-1K→LV II	Accuracy	**0.8557** 0.5114	0.8358 0.5570	0.8159 0.5823	0.8209 0.6329	0.8507 0.6835	0.8408 **0.8101**
	Precision	**0.7814** 0.5430	0.7216 0.5437	0.7737 0.5689	0.7866 0.6191	**0.8676** 0.6696	0.8136 **0.8187**
	Recall	**0.8899** 0.5013	0.8276 0.5556	0.8088 0.5806	0.8397 0.6417	0.8719 0.6986	0.8895 **0.7958**
	F1-score	0.8246 0.5132	0.7544 0.5464	0.7880 0.5676	0.8068 0.6216	**0.8626** 0.6721	0.8448 **0.8003**
	MCC-score	**0.7633** 0.3145	0.7286 0.3881	0.6857 0.4285	0.6951 0.5001	0.7477 0.5714	0.7445 **0.7357**

**Table 5:** Statistical analysis of classification performance metrics (mean ± std) across multiple random seeds on cross-domain tasks. Note: upper row represents source domain result, lower row represents target domain result.

Task	Accuracy	Precision	Recall	F1-Score	MCC-Score
MGD-1K→K5M	0.8388 ± 0.0051 0.7793 ± 0.0169	0.7979 ± 0.0099 0.7816 ± 0.0341	0.8497 ± 0.0157 0.7769 ± 0.0298	0.8175 ± 0.0051 0.7686 ± 0.0193	0.7330 ± 0.0088 0.6971 ± 0.0235
MGD-1K→CR-2	0.7920 ± 0.0058 0.7486 ± 0.0114	0.7440 ± 0.0049 0.7009 ± 0.0142	0.8200 ± 0.0151 0.7720 ± 0.0405	0.7720 ± 0.0029 0.7248 ± 0.0221	0.6635 ± 0.0092 0.6176 ± 0.0188
MGD-1K→LV II	0.8428 ± 0.0024 0.8177 ± 0.0179	0.8038 ± 0.0120 0.8020 ± 0.0202	0.8903 ± 0.0060 0.8003 ± 0.0175	0.8398 ± 0.0076 0.7988 ± 0.0174	0.7451 ± 0.0052 0.7366 ± 0.0247

## Data Availability

The publicly available data presented in this study are openly available in MGD-1K at https://mgd1k.github.io/index.html (accessed on 20 February 2025). The real-world clinical dataset contains patient information and is therefore not publicly available due to privacy and ethical restrictions. The data are held by the authors and may be made available upon reasonable request to corresponding authors.

## Abbreviations

The following abbreviations are used in this manuscript:

MGD	Meibomian Gland Dysfunction
DL	Deep Learning
MG	Meibomian Gland
DANN	Domain-Adversarial Neural Network
UDA	Unsupervised Domain Adaptation
MCC-score	Matthews correlation coefficient
ML	Machine Learning
SVM	Support Vector Machine
ROI	Regions of Interest
XAI	Explainable Artificial Intelligence
DA	Domain adaptation
SNR	Signal-to-Noise Ratio
ADAM-Net	Attention-guided Domain-Adaptive Multi-task Network
SGSA	Segmentation-Guided Spatial Attention
GRL	Gradient Reversal Layer
CBAM	Convolutional Block Attention Module
MSE	Mean Squared Error
BCE	Binary Cross-Entropy
t-SNE	t-distributed Stochastic Neighbor Embedding
IoU	Intersection over Union
CDAN	Conditional Domain Adaptation Network
DAN	Deep Adaptation Network
JAN	Joint Adaptation Network
MDD	Margin Disparity Discrepancy
BSP	Batch Spectral Penalization
MCC	Minimum Class Confusion
Grad-CAM	Gradient-weighted Class Activation Mapping
GT	Ground Truth
Pred	Predicted
